# Harvested Wood Products as a Carbon Sink in China, 1900–2016

**DOI:** 10.3390/ijerph16030445

**Published:** 2019-02-02

**Authors:** Luyang Zhang, Yankun Sun, Tianyuan Song, Jiaqi Xu

**Affiliations:** College of Resources and Environment, Northeast Agricultural University, 600 Changjiang Road, Harbin 150030, China; zly@neau.edu.cn (L.Z.); tian-yuan1023@163.com (T.S.); kevin8257@163.com (J.X.)

**Keywords:** climate change mitigation, harvested wood products, solid waste disposal site, carbon reporting and accounting, China carbon budget

## Abstract

The use of harvested wood products (HWPs) influences the carbon flux. China is both the major producer and trader of HWP, so estimating the carbon stock change of China’s HWP is important to help curb climate change. Accurate reporting and accounting of carbon flows in the HWP pool is needed to meet greenhouse gas monitoring and climate change mitigation objectives under the United Nations Framework Convention on Climate Change (UNFCCC) and the Paris Agreement. This study applied production approach (PA) to estimate the carbon stock change of China’s HWP from 1900 to 2016. During the estimating period, the carbon stock of HWP in use and deposed at solid waste disposal sites (SWDS) were 649.2 Teragrams Carbon (TgC) (346.8 TgC in wood-based panels, 216.7 TgC in sawnwood and 85.7 TgC in paper & paperboard) and 72.6 TgC, respectively. The carbon amount of annual domestic harvest HWP varied between 87.6 and 118.7 TgC. However, the imported carbon inflow increased significantly after the 1990s and reached 47.6 TgC in 2016, accounting for 46% of the domestic harvest of that year. China has great mitigation potential from HWP and use of this resource should be considered in future strategies to address climate change.

## 1. Introduction

Forests function as carbon sinks and stores, with the potential to mitigate climate change. When trees are harvested, the Harvested Wood Products (HWP) are an extension of forest resources and are part of the forest’s carbon cycle. Fixed carbon transfers from forests to HWPs through deforestation and there is a lag in carbon emissions before the consumption of wood products. The worldwide annual volume of wood removal has increased from approximately 2.75 billion m^3^/yr to 3.0 billion m^3^/yr from 1990 to 2011 [1]. The roundwood production was around 3.8 billion m^3^ in 2017, which has increased by 21% compared with the amount in 1980 [2]. According to the second assessment report of the Intergovernmental Panel on Climate Change (IPCC) [3], the annual global increment of carbon stock in HWPs was around 26 Mt/yr (1 Mt = 10^6^ t). Others estimated larger increments between 40 Mt to 140 Mt per year [4,5].

Under the United Nations Framework Convention on Climate Change (UNFCCC), signatory countries have the responsibility to report the change in carbon stock in the Agriculture, Forestry and Other Land Use sector (AFOLU) using methods described in the IPCC Guidelines and Good Practice Guidance methods [6,7,8]. Annex I (developed) countries have stronger reporting expectations than non-Annex I (developing) countries, including the use of more sophisticated (higher tier) methods and more complete reporting of different pools, including HWP. The IPCC describes various different methodological approaches to estimate and report carbon fluxes from HWPs, including the stock-change approach (SCA), the production approach (PA) and the atmospheric-flow approach (AFA). The Conference of the Parties (COP) 17 of the UNFCCC held in Durban in 2011 included decision (2/CMP.7) which made accounting of HWP mandatory. It also specified that “accounting of HWP is confined to products in use where the wood was derived from domestic harvest,” meaning that only domestically produced and harvested HWP should be included in national accounting [8]. For this reason, the subsequent *2013 Revised Supplementary Methods and Good Practice Guidance Arising from the Kyoto Protocol* [8] (hereinafter referred to as the *2013 Supplementary*) only included methods using the PA method for the second commitment period of the Kyoto Protocol.

China plays a highly significant role in both global wood production and consumption due to its rapid economic development. In 2016, China’s consumption of sawnwood surpassed the USA to become highest in the world. China has been the largest producer and consumer of wood-based panels and paper products for years [9]. China also plays an important role in international trade of wood products. China is the largest roundwood importer and the largest exporter of wood-based panels [9,10]. In 2016, about 20% of China’s industrial roundwood consumption was satisfied by imports based on the statistics of FAOSTAT (Food and Agriculture Organization Statistical Databases). Production and consumption of main forest products in China have grown faster than in the rest of the world. Despite the recent slow growth of China’s overall economy, the nation’s high demand for timber is likely to sustain growth and timber imports are expected to increase by 60 million m^3^ or 12–13% of the world total harvest by 2025 [11]. The domestic deficit of wood products is expected to increase in the future due to the new logging ban in the national forests of China [10]. These changes will influence contributions to the HWP carbon pool when using the default IPCC estimation methods (PA).

The carbon pool in HWP of China represents a continuous increasing carbon sink [12,13,14,15]. Yang et al. [13] and Ji et al. [14] estimated the carbon stock in China’s HWP by using the latest PA approach, as recommend by the IPCC. Both studies employed the data from FAOSTAT but reached different results. The cumulative carbon stock by 2014 was estimated as 705.6 Teragrams Carbon (TgC) by Ji et al. [14], which was significantly higher than the estimation by Yang et al.’s [13] of only 473.3 TgC in 2015. Both studies ignored the stock change in retired wood products. Zhang et al. [15] performed a more comprehensive evaluation including HWP in use, discarded mill residue, wood fiber input and waste HWP, by using data obtained from China Forestry Statistical Yearbook and the SCA estimation approach. In their study, the total cumulative carbon stock from 1900–2015 was 2195.2 TgC. As a large importing country, SCA is the favorable estimating method of China’s greenhouse gas inventories in terms of the contribution of HWP but SCA is no longer an accepted approach for a reporting country.

Currently, China’s HWP carbon stock including wood products in use and after use calculated by PA approach has not yet been clarified and the carbon balance of HWP has not included in China’s national greenhouse gas inventories. So, the mitigation from HWP carbon pool in China remains a question. Therefore, the goal of this study was to estimate the carbon sequestration and emissions of HWP in China by PA, including estimation of annual carbon stock changes and retired HWP waste from 1900 to 2016. We also analyzed the HWP carbon pool structure and explored the relevant policies influencing the carbon pool of HWP in China and make recommendations for future mitigation policy.

## 2. Materials and Methods

### 2.1. Annual Change in Carbon Stock in “Products in Use”

The methodology of estimation of carbon removals and emissions in HWP was first described in the *2006 IPCC Guidelines for National Greenhouse Gas Inventories* [16] (referred to as *2006 Guidelines* hereafter) (Vol. 4 Ch. 12). SCA, AFA and PA are the main recommended methods. In the *2013 Supplementary* [8], the PA method was revised based on the *2006 Guidelines* and became the only accepted approach for reporting countries. The estimation methods in our study are the revised PA methods described briefly below.

The first-order decay method is employed to estimate the carbon change in HWP. Equations (1) and (2) estimate the carbon stock and annual change in the HWP pool from production of the domestic harvest.

(1) $$C\left(i+1\right){=e}^{-k}\cdot C\left(i\right)+[\left(1-e-k\right)k]\cdot Inflow(i)$$

(2) ΔC(i)=C(i+1)−C(i),

where *C*(*i*) = the carbon stock at the beginning year of *i*, *C* (1900) = 0.

*K* = *ln* (2)/*HL*. *K* represents the decay constant of first-order decay and is given in units, yr^−1^. *HL* is the half-life of the HWP pool in years. A half-life is the number of years it takes to lose one-half of the material currently in the carbon pool [17]. Half-life years are 25, 35 and 2 years for wood-based panels, sawnwood and paper products followed the IPCC default values.

(3) $$Inflow\left(i\right){=P\times f}_{DP}\left(i\right)$$

(4) $$f_{DP}\left(i\right)=\{\begin{array}{c} f_{IRW}\left(i\right),\;for\;\mathrm{sawnwood}\;\mathrm{and}\;\text{wood-based}\;\mathrm{panels}\\ f_{\mathrm{IRW}}\left(i\right)\cdot f_{\mathrm{PULP}}\left(i\right),\;\mathrm{for}\;\mathrm{paper}\;\mathrm{and}\;\mathrm{paperboard} \end{array}$$

(5) $$f_{\mathrm{IRW}}\left(i\right)=\frac{{\mathrm{IRW}}_{P}\left(i\right)-{\mathrm{IRW}}_{\mathrm{EX}}\left(i\right)}{{\mathrm{IRW}}_{P}\left(i\right){+\mathrm{IRW}}_{\mathrm{IM}}\left(i\right){+\mathrm{IRW}}_{\mathrm{EX}}\left(i\right)}$$

(6) $$f_{\mathrm{PULP}}\left(i\right)=\frac{{\mathrm{PULP}}_{P}\left(i\right){-\mathrm{PULP}}_{\mathrm{EX}}\left(i\right)}{{\mathrm{PULP}}_{P}\left(i\right){+\mathrm{PULP}}_{\mathrm{IM}}\left(i\right){-\mathrm{PULP}}_{\mathrm{EX}}\left(i\right)}$$

*Inflow*(*i*) = the inflow to the HWP pool during year *i*. Δ*C*(*i*) = carbon stock change of the HWP pool during year *i*. *P* = carbon in annual production of solid wood or paper products of the country, *IM* = imports, *EX* = exports, *IRW* = industrial roundwood, *PULP* = paper & paperboard. The Equation (7) calculates the extrapolated growth between 1900 and 1960, because the data were only available from 1961 in FAOSTAT.

(7) $$V_{t}{=V}_{1961}\cdot e^{(U\cdot \left(t-1961\right))},$$

where *t* is year;

*V_t_* is the annual amount of solidwood or paper product from production, imports or exports;

*U* is estimated annual rates of change for industrial roundwood consumption of Asia between 1900 and 1961, yr^−1^ (*U* = 0.0217).

Annual domestic harvest: *H* = *IRW_H_* × BF + Fuelwood, where BF = bark factor (BF = 1.13 follows the IPCC default value).

### 2.2. Annual Change in Carbon Stock in Solid Waste Disposal Sites (SWDS)

The estimation methodology of carbon removals and emissions of retired HWP in SWDS follows the recommendation in the *2006 Guidelines* (Vol. 5 Ch. 3) [16]. The carbon decay in SWDS is calculated by first-order decay method.

Equation (8) represents the mass of Decomposable Degradable Organic Carbon (DDOC) that remains not decomposed at the end of the I year in SWDS and the mass of DDOC decomposed into CH_4_ and CO_2_ as shown in Equation (9).

(8) $${\mathrm{DDOC}}_{m}\left(i+1\right){=\mathrm{DDOC}}_{m}\left(i\right)\times e^{-\mathrm{kt}},$$

(9) $${\mathrm{DDOC}}_{\mathrm{mdecomp}}\left(i+1\right){=\mathrm{DDOC}}_{m}\left(i\right)\times (1-e^{-\mathrm{kt}}),$$

where: *DDOC_m_* = mass of degradable organic carbon (DOC) in the disposal site at time *t*, *k* = decay rate constant in y^−1^ which vary from climate type and different waste. Most of China belongs to the north temperate zone and half of China is wet and half is dry climate [18], therefore we choose a *k* value for paper waste of 0.025 and wood waste of 0.05 based on *2006 Guidelines*. *K* determines the half-life time (*t*_1/2_) in the reaction, the time required to decrease by 50% the amount. *k* = *ln* (2)/*t*_1/2_ (y^−1^). In this condition, half-life times are 27.7 years and 13.9 years for paper waste and wood waste, respectively.

The *DDOC_m_* from the amount of waste disposed is calculated as Equation (10).

(10) $${\mathrm{DDOC}}_{{\mathrm{md}}_{T}}{=W}_{T}\cdot \mathrm{DOC}\cdot {\mathrm{DOC}}_{f}\cdot \mathrm{MCF},$$

where: *DDOC_mdT_* = *DDOC_m_* disposed in year *T*, *W_T_* = mass of waste disposed in year *T*, *DOC* = fraction of degradable organic carbon in disposal year, *DOC_f_* = fraction of *DOC* that can decompose in the anaerobic conditions in the SWDS, *MCF* = CH_4_ correction factor.

Equation (11) calculates the amount of CH_4_ generated from decomposition in SWDS. In China there is no methane collection [19,20] and we also assume no oxidation during the emission. The amount of CH_4_ emitted is the same as the amount generated.

(11) $${\mathrm{CH}}_{4}{\mathrm{generated}}_{T}{=\mathrm{DDOC}}_{m}{\mathrm{decomp}}_{T}\cdot F\cdot 16/12,$$

where: *CH_4_generated_T_* = the amount of CH_4_ generated from the *DDOC_m_* that decomposes, *DDOC_m_decomp_T_* = *DDOC_m_* decomposed in year *T*, *F* = fraction of CH_4_, by volume, in generated landfill gas.

Some of the *DOC_m_* in waste will not decay into CH_4_ or CO_2_ and this part of carbon will be stored long-term in the SWDS. The annual change of long-term stored carbon can calculate by Equation (12) below.

(12) $${\mathrm{DOC}}_{m}{\text{long-term}\;\mathrm{stored}}_{T}{=W}_{T}\cdot \mathrm{DOC}\cdot {(1-\mathrm{DOC}}_{f})\cdot \mathrm{MCF},$$

*DOC_f_* = the proportion of disposed *DOC_m_* that will remain in SWDS for long term, default value is 50%.

### 2.3. HWP Contributes to Carbon Removals and Emissions

The annual contribution of HWP is equal to the annual change in HWP carbon stock. Equation (13) below estimates the removals and emissions from the carbon pool in HWP using the PA.

(13) $$\Delta C_{\mathrm{PA}}=\Delta C_{\mathrm{HWP}\_\mathrm{DH}}+\Delta C_{\mathrm{SWDS}\_\mathrm{DH}}$$

Annual change in carbon stock in PA (∆*C_PA_*) includes a) HWP in use (∆*C_HWP_DH_*) and b) HWP in SWDS (∆*C_SWDS_DH_*) for products made from wood domestically harvested in the reporting country; this includes exported HWP to other countries.

### 2.4. Data Sources

The UNFCCC defines HWP as wood materials and products harvested from forests [17], materials that could be used as fuel or industrial materials. The HWP carbon pool includes:

1. Solid wood products including sawnwood and wood-based panels, and
2. Paper products, such as paper & paperboard.

The carbon flow in the forest product trade includes wood pulp and recovered paper, industrial roundwood, chips and particles, wood charcoal and wood residues. All HWP data used in this analysis are derived from the Forestry Production and Trade in the FAOSTAT database (http://www.fao.org/faostat/en/#data/FO). The production of HWP refers to the production in mainland China. Table 1 presents the carbon conversion factors for estimating the carbon contained in different HWPs. Because we need a base year to calculate the carbon budget in a given time and 1900 is the recommendation from UNFCCC. The FAOSTAT database has statistics from 1961 onwards, so the estimates from 1900–1960 were extrapolated by using Equation (7). Therefore, our analysis focused on the results after 1961 based on the real HWP data. The previous researchers [13,14,21,22] also did the discussion and analysis after 1961 but calculated the carbon budget from 1900.

The disposal options of HWP after use are sourced from the China Circular Economy Yearbooks [20,23], Cai et al. [19] and Zhang et al. [15]. Table 2 shows the proportion of discarded wood products by different disposal methods from 1900–2016. In China, three treatments are used for the waste of wood products: combustion, landfill and open dump. Open dump was the main option before 1980 but with increased management of waste, no waste is treated as open dump since 2007. Most retired wood products were disposed in a landfill in recent years and others were burned directly. Open dump and landfill correspond to unmanaged, shallow and unmanaged, deep, respectively, in the SWDS models.

## 3. Results

### 3.1. Carbon Stock and Its Annual Change Stored in HWP

**Figure 1:**
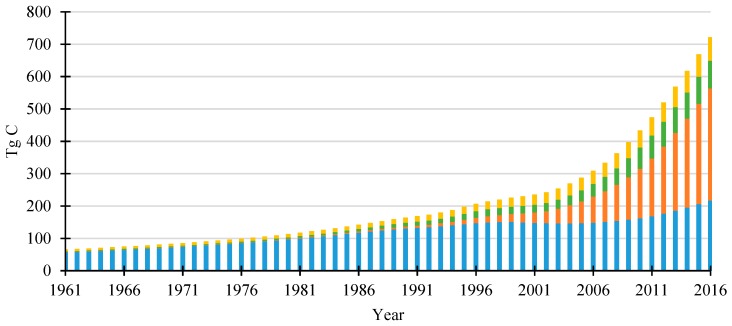
Carbon stored in HWP in China by different categories.

**Figure 2:**
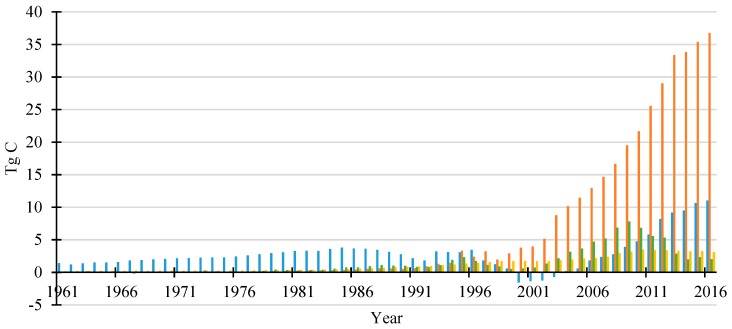
Annual change of carbon stored in HWP in china by different categories.

An overview of carbon stock accumulated in main HWP categories from 1961 to 2016 in China is provided in Figure 1. The amount of carbon in the HWP cool was estimated since 1900 but the results between 1900 and 1960 were extrapolated by Equation (7), therefore we only analyzed the data from 1961 based on FAOSTAT. Continued growth was the significant characteristic of China’s HWP carbon pool. The total amount of carbon stock increased from 65.9 TgC to 721.9 TgC from 1961 to 2016 and there was increased annual stock change in recent years. The accumulated carbon stock amounts were 15.0, 26.4, 45.5, 61.6, 167.1 and 287.9 TgC in 1960s, 1970s, 1980s, 1990s, 2000s and 2010–2016, respectively. It is very obvious that the carbon accumulation increased over time. The annual carbon stock change varies between different HWP categories, as represented in both Figure 1 and Figure 2. Sawnwood was the main carbon stock contribution in early years and then was surpassed by wood-based panels in 1995. During 2000–2003, there was no carbon stored from sawnwood and the annual carbon changes were negative (−1.2 TgC on average). The carbon stock change of sawnwood showed a stable increasing trend after 2003 and was 11.0 TgC in 2016. The wood-based panels provided the dominant carbon accumulation in recent years and the annual stock change of wood-based panels increased to 36.8 TgC in 2016, 20 times larger than the amount in the 1990s. The contribution from paper & paperboard was relatively small and showed high fluctuations. The carbon stock for paper has been declining since a peak in 2009 (7.8 TgC) and carbon stock from SWDS has showed steady growth from 0.2 TgC to 3.5 TgC annually between 1961 and 2010. However, the annual carbon stock from SWDS has decreased to 3.1 TgC in 2016 due to the increasing proportion of combustion of waste wood products.

**Figure 3:**
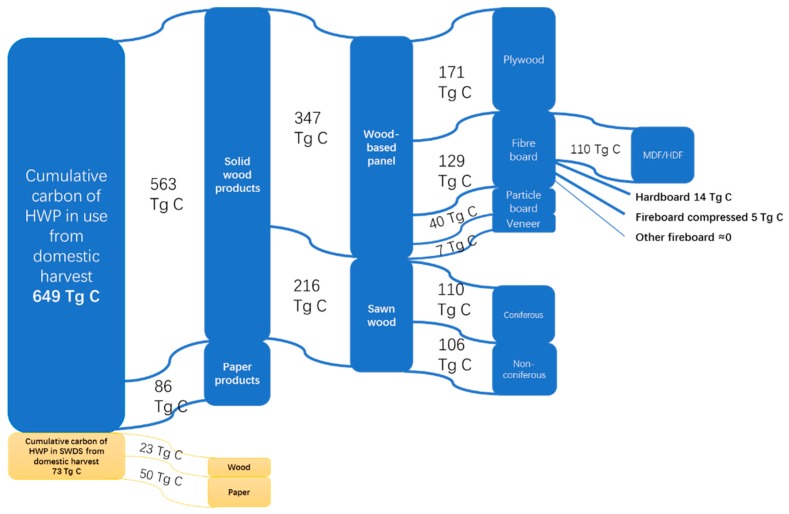
Carbon contribution by different categories including HWP in use and waste from domestic harvest in China, the period of cumulative carbon stock is from 1900 to 2016.

The data presented in Figure 3 shows the carbon contributions from different wood products in use and waste. Solidwood products were the main source of carbon sequestration in use and more carbon stock came from paper in SWDS. Because solidwood products have longer lifespans than paper products, more solidwood products maintain carbon during usage and more paper goes to SWDS. The carbon accumulation from wood-based panels was the major component of solidwood products in China, 131 TgC more than sawnwood. In this category, 86% of carbon stock was sourced from plywood and fiberboard products and MDF/HDF was the leading item among fiberboard products. The distribution of coniferous and non-coniferous in sawnwood products was basically equal.

### 3.2. Carbon Flow in Roundwood Production, Imports and Exports in China

**Figure 4:**
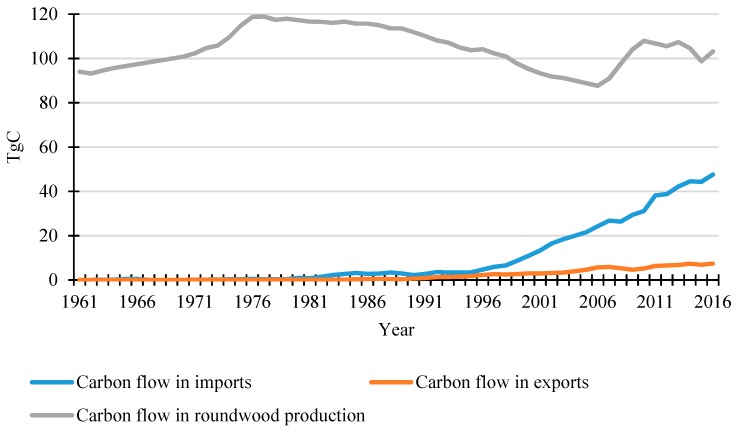
Carbon flow in domestic roundwood production, imports and exports in China.

**Figure 5:**
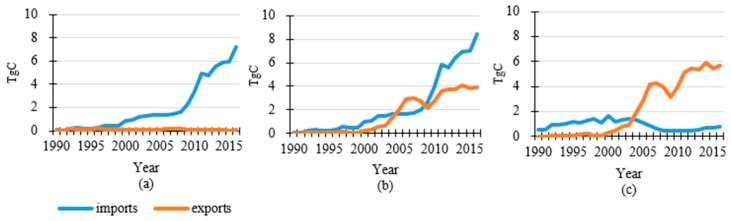
Trading carbon flow of semi-products in China. (**a**) Description of trading carbon flow of sawnwood in China; (**b**) Description of trading carbon flow of wood-based panels in China; (**c**) Description of trading carbon flow of paper & paperboard in China.

Figure 4 displays the carbon flow in domestic roundwood production and imports and exports from 1961 to 2016 in China. The average annual domestic roundwood harvest was 104.5 TgC over the 56 years studied here. The domestic harvest was 94.0 TgC at 1961 and peaked between 1976 and 1986 with an annual harvest around 115 TgC, then gradually decreased to 87.6 TgC in 2006. Although the amount recovered to 103.2 TgC in 2016 with some fluctuation, this was still lower than the average for the whole time period. In contrast, the carbon inflow from importing increased dramatically, especially after the mid-90s. The carbon inflow from importing was quite small before mid-90s (3.5 TgC in 1995) but then soared to 47.6 TgC in 2016 with a mean value of 36.9 TgC during the last decade. The increase of carbon inflow from exporting was much smaller during this time. A significant climb was observed for the most recent 15 years and the value increased from 3.0 TgC in 2000 to 7.4 TgC in 2016. Compared with importing, the exporting carbon inflow only accounted for around 16% in the most recent five years. The different trade patterns were examined for sawnwood, wood-based panels and paper & paperboard (Figure 5). From 1990 to 2016, the carbon flow of sawnwood and wood-based panel imports showed a significant increase, which was opposite from the trend for paper & paperboard. In 2016, the importing carbon flows of sawnwood and wood-based panels were 7.2 TgC and 8.5 TgC, respectively. Importing carbon flow from paper & paperboard peaked around 2000 (1.6 TgC), decreased to 0.4 TgC in 2009 and then recovered to 0.8 TgC in 2016. In terms of exports, the small amount of sawnwood was negligible during the whole period. The exporting carbon flow from wood-based panels showed a considerable increase to 3.9 TgC in 2016 and exceeded imports during 2004–2009. The exporting carbon flow from paper & paperboard overtook the imports in 2004 and increased to 5.7 TgC in 2016, 7 times larger than the amount from imports. Obviously, wood-based panels played an important role in both imports and exports in China.

### 3.3. Carbon Emissions from HWP after Use

**Figure 6:**
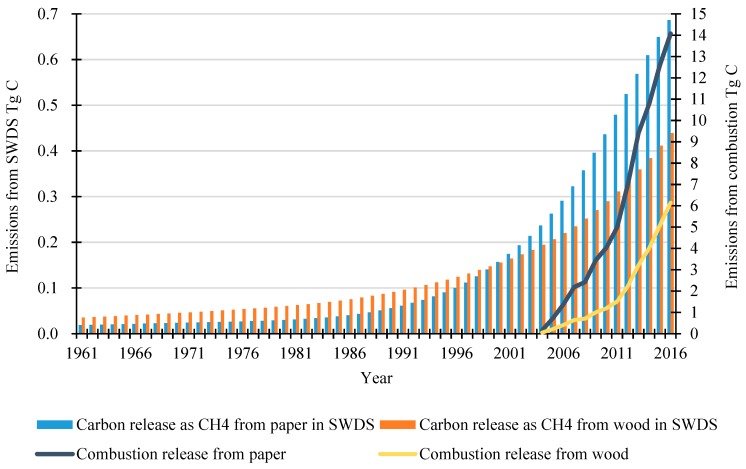
HWP carbon emissions after use.

The disposal method greatly influences the carbon emissions from the HWP pool after use. The HWP waste discarded in SWDS emits methane as the main carbon release. As we can see from Figure 6, the carbon released as methane from paper waste made up a larger proportion than wood waste after 1999 and increased faster than wood. Until 2016, the carbon emissions released as CH_4_ from paper and wood waste were 0.7 and 0.4 TgC, respectively and both showed a steady rising trend during the estimating period. Before 2004, no HWP waste was burned directly. However, the emissions from combustion increased as combustion became a more popular disposal method, with over 30% of the waste disposed by combustion after 2014. This method releases all carbon into the atmosphere immediately, losing the opportunity to sequestrate carbon in SWDS. As a result, this method has greater carbon emissions compared with discarding into SWDS. In 2016, 14.1 and 6.1 TgC of carbon was directly released from paper and wood waste by combustion. 

## 4. Discussion

### 4.1. Impacts of the Estimation Approach and Implications for Policy

As a major timber importing country, the SCA is the best method for China to calculate and report contributions to UNFCCC, since the importing of wood products increases the carbon stock inside China. However, the current PA does not consider trade in the estimation methods. If the domestic harvest production declines in China due to a logging ban, the reported contribution of HWP may weaken if calculated by the PA despite a future increasing HWP supply from importing. The PA encourages the use of a nation’s HWP exports to increase the carbon stock. However, tracing exported HWP is difficult. In this approach, it is assumed that exported HWP are consumed in the same way as they are in the domestic country but this may not be a valid assumption [24,25]. Moreover, the exporting countries have the responsibility to report any carbon emissions in their national HWP pool, so importing countries may be unwilling to effectively manage imported HWP, which could consequently lessen service lifetime. That conflicts with the mitigating intention and may also result in a large error when estimating the overall carbon removal for reporting countries. An advantage of the production approach is that it is easy to distinguish the source of the HWP. Practices including forest management, forest degradation and deforestation are all based on the origin of HWPs [26]. Although the SCA more completely considers the exports and imports of HWP, it does not address the sources of HWPs. The AFA is more suitable to account for carbon emissions, such as emissions from the energy sector and the carbon pool of HWP acts as a carbon sink rather than as a carbon source in most cases. To enhance the accuracy of the estimation of production approach, additional studies tracing he carbon flow change in HWP cross-border transactions are needed.

With an increasing wood product demand, China may require more imports after the logging ban, as mentioned in the introduction. Currently, HWP exports to China from Africa accounts for more than 75% of its total timber exports [27]. The forest management in African countries is greatly influenced by China. As described above, China has no responsibility to report imported HWP according to the production approach but if China neglects the management of those imported wood products, more carbon leakage would result. Additionally, the potential of carbon sequestration from retired HWP deserve more attention. Burning without energy recovery is the worst disposal treatment but it has exhibited a rising trend in China.

### 4.2. Comparison with Previous Studies

There are two reliable original data sources of China’s HWP: FAOSTAT and the China Forestry Statistical Yearbooks. However, the classification of HWP in the China Forestry Statistical Yearbooks does not match with that used by the UNFCCC and does not include timber trading data. Therefore, like this study, most studies have used FAOSTAT as the source of original data. Even though our data sources are the same, the estimated carbon stocks determined in our study are smaller [12,13,14]. There are two main reasons for these differences. First, most previous studies only followed the *2006 Guidelines* and included other industrial wood products. However, the *2013 Supplement* excludes some of these items and the FAOSTAT data for other industrial wood products are not as accurate as the other data [16]. Second, the choice of parameters for HWP can significantly affect the calculations. Some studies used all the default factors from IPCC but others have tried to consider the specifics of China’s condition. The *2013 Revised Supplement* suggested that the latest carbon conversion factors are much smaller than the factors used in the *2006 Guidelines*. Zhang et al. [15] used the HWP data from China Forestry Statistical Yearbooks and estimated HWP carbon stock in use as 895.6 TgC and the amount disposed by SWDS as 179.7 in 2015. They included “direct use” of industrial roundwood, which was not considered in our study. The carbon stock of “direct use” of industrial roundwood was 66% to 20% of the solid HWP from 1950 to 2015 in their study. As we use different HWP data sources, the cumulative carbon stock was smaller than the results from Zhang et al. [15]. Because of the different data used, for the analysis up to 2015, the sawnwood, wood-based panels and paper & paperboard were 10%, 20% and 27% less in our study.

### 4.3. Limitations

Uncertainties in this work generally result from three aspects. First, there may be error in the original production data of HWP and the carbon conversion factors. In FAOSTAT, the trading data before the 1990s are missing, so in this study, these have been set to zero. Another potential factor is that this study ignored recycled HWP but this material may extend the carbon storage in products. Third, the statistical data on finished HWP in China are scarce, so the carbon flow of semi-finished HWP to SWDS is not clear.

## 5. Conclusions

This study investigated the mechanisms of carbon sequestration, carbon emissions and removals associated with carbon flow in the HWP pool in China. By 2016 in China, the carbon stock in HWP in use was 649.2 TgC and the disposed amount was 72.6 TgC. China’s HWP pool can serve as a significant carbon sink. With increased consumption, the rate of carbon pool accumulation also surged during the estimating period and the acceleration of carbon accumulation may continue. That means increasing carbon pool of HWP could buy time to cope with climate change. On the other hand, the use of wood-based panels has the greatest impact on China’s HWP carbon sequestration in the products in use section. The trading carbon flow from wood-based panels was also the main part affecting China’s HWP pool. In addition, it should be noticed that the increasing emissions caused by combustion after use. With China’s current logging ban and growing timber demands, the importing carbon flow would increase. However, the PA would underestimate the carbon sink of China’s HWP pool. To promote the mitigation efforts from China’s HWP, more attention should be paid to reduce the amount of burning of the wasted HWPs and enhance the management of the wood-based panels.

## Figures and Tables

**Table 1:** Carbon conversion factors in HWPs used in the models.

HWP Categories	C Conversion Factor
	(per air dry volume) [Mg C/m^3^]
Sawnwood (aggregate)	0.229
Coniferous sawnwood	0.225
Non-coniferous sawnwood	0.28
Wood-based panels (aggregate)	0.269
Hardboard	0.335
Other fiberboard	0.075
Fiberboard compressed	0.315
MDF/HDF ^1^	0.315
Particle board	0.269
Plywood	0.267
Veneer sheets	0.253
	(per air dry mass) [Mg C/Mg]
Paper & paperboard (aggregate)	0.386
Charcoal	0.765
Roundwood (aggregate, Industrial roundwood, pulpwood, chips, particles, wood fuel, wood residues ^2^	0.26
Coniferous roundwood	0.225
Non-coniferous roundwood	0.295

^1^ Assume half MDF (Medium Density Fiberboard) and half HDF (High Density Fiberboard). ^2^ Assume half temperate species and half tropical species.

**Table 2:** Different disposal options of waste HWP after use.

Year	Combustion	Unmanaged, Shallow	Unmanaged, Deep
1900–1980	0%	100%	0%
1981	0%	97%	3%
1982	0%	93%	7%
1983	0%	90%	10%
1984	0%	87%	13%
1985	0%	84%	16%
1986	0%	80%	20%
1987	0%	77%	23%
1988	0%	74%	26%
1989	0%	70%	30%
1990	0%	67%	33%
1991	0%	64%	36%
1992	0%	60%	40%
1993	0%	57%	43%
1994	0%	54%	46%
1995	0%	51%	49%
1996	0%	47%	53%
1997	0%	44%	56%
1998	0%	41%	59%
1999	0%	37%	63%
2000	0%	34%	66%
2001	0%	31%	69%
2002	0%	28%	72%
2003	0%	24%	76%
2004	1%	19%	80%
2005	4%	13%	83%
2006	7%	7%	87%
2007	10%	0%	90%
2008	10%	0%	90%
2009	13%	0%	87%
2010	14%	0%	86%
2011	16%	0%	84%
2012	21%	0%	79%
2013	27%	0%	73%
2014	30%	0%	70%
2015	34%	0%	66%
2016	37%	0%	63%
